# Effects of Add-On Ultramicronized N-Palmitol Ethanol Amide in Patients Suffering of Migraine With Aura: A Pilot Study

**DOI:** 10.3389/fneur.2018.00674

**Published:** 2018-08-17

**Authors:** Domenico Chirchiglia, Erika Cione, Maria C. Caroleo, Minyan Wang, Giulio Di Mizio, Noemi Faedda, Teodosio Giacolini, Serena Siviglia, Vincenzo Guidetti, Luca Gallelli

**Affiliations:** ^1^Department of Medical and Surgical Science, School of Medicine, University of Catanzaro, Catanzaro, Italy; ^2^Department of Pharmacy Health and Nutritional Sciences, University of Calabria, Rende, Italy; ^3^Department of Biological Sciences, Centre for Neuroscience, Xi'an Jiaotong-Liverpool University, Suzhou, China; ^4^Department of Law, Ecenomy and Sociology, University of Catanzaro, Catanzaro, Italy; ^5^Section of Child and Adolescent Neuropsychiatry, Department of Human Neuroscience, “Sapienza” University, Rome, Italy; ^6^Clinical Pharmacology and Pharmacovigilance Operative Unit, Department of Health Science, University of Catanzaro, Mater Domini Hospital Catanzaro, Catanzaro, Italy

**Keywords:** Migraine with aura, ultramicronized palmitoyl ethanol amide, pain, clinical trial, efficacy, safety

## Abstract

**Background:** Palmitoyl ethanol amide (PEA) is an endogenously produced substance showing anti-nociceptive effect through both receptor and non-receptor mediated effects at the level of different cellular and tissue sites. This study showed the results of a single blind study that was conducted to evaluate both the safety and the efficacy of ultramicronized PEA (umPEA; 1,200 mg/day) for up 90 days in patients suffering of Migraine with Aura (MA) treated with NSAIDs.

**Methods:** A total of 20 patients, 8 male (33–56-years, average 41.4 ± 7.8) and 12 female (19–61-years, average 38.5 ± 11.9) with MA were admitted to our observation and diagnosed according to ICHD-3 criteria, they received umPEA (1,200 mg/day) in combination with NSAIDs for up to 90 days. They were revaluated at 30, 60, and 90 days after treatment.

**Results:** umPEA administration induced a statistically significant and time dependent pain relief. In particular, these effects were evident at 60 days (male *P* = 0.01189; female *P* = <0.01) and they lasted until the end of the study (male *P* = 0.0066; female *P* = 0.01473).

**Conclusion:** Although further studies are needed, our findings indicate that in patients suffering of MA treatment with umPEA had good efficacy and safety which candidate this compound as a therapeutic tool in pain migraine management.

## Introduction

Migraine is a common disabling primary headache disorder. It is the sixth highest cause of years lost due to disability worldwide (1, 2) with high prevalence in young adults (3, 4). Migraine can be classified in two major types: Migraine without aura characterized by headache with specific features and associated symptoms and Migraine with aura (MA) characterized by the transient focal neurological symptoms that usually precede or sometimes accompany the headache (5).

In MA, the word “aura” denotes recurrent attacks of reversible neurologic symptoms (e.g., visual, sensory, speech, motor, or other central nervous symptoms) usually lasting few minutes. Often the symptoms are unilateral, occurring on only one side of the body or of the visual field; the aura is generally followed by a headache (5).

Traditionally migraine treatment (with or without aura) includes both prophylactic therapy, aimed at reducing the frequency and severity of attacks, and acute therapy for halting the progression of attacks. Unfortunately prophylactic therapy rarely eliminates migraine (6), even though it is effective in improving responsiveness to acute therapy, thus ameliorating the level of disability. Triptans, non-steroidal anti-inflammatory drugs (NSAIDs), and antiemetics represent the mainstay of acute therapy (7, 8); however, in MA pain is less responsive to triptans (9) and NSAIDs use is hampered by the development of several adverse drug reactions (ADRs) (10, 11). Other drugs such as antidepressants (duloxetine and amitriptyline) and anticonvulsants (e.g., pregabalin and gabapentin) are also able to induce pain relief through the modulation of synaptic neurotransmitter levels leading to an improvement of quality of life (12). However, like NSAIDs their use is limited by the development of heavy side effects. Palmitoyl ethanol amide (PEA) is an endogenous fatty acid amide widely distributed in different tissues, including nervous tissues; it is synthesized on demand. PEA is emerging as a novel therapeutic approach in pain and inflammatory conditions (13). PEA has been reported to be effective in animal models of chronic pain and inflammation as well as in several clinical trials on various pain states (14–17). However, to date no studies have been performed to evaluate the role of PEA in the management of MA.

The aim of this study was to evaluate the efficacy and the safety of chronic administration of ultramicronized PEA (um-PEA) in patients with MA treated with NSAIDs.

## Methods

### Study

We performed a prospective single-blind study from 2014 to 2015 in patients admitted to the Neurosurgery Division of “Mater Domini” University Hospital in Catanzaro. The study protocol was approved by the Local Ethics Committee (Catanzaro Centro protocol number 235/2017), the enrolled patients signed the written informed consent, and the work was conducted in compliance with the Institutional Review Board/Human Subjects Research Committee requirements. In order to exclude any risk for the patients, both patients and physicians that evaluated the patients knew the protocol and the group of treatment, while physicians that evaluated the data were blinded to both protocol and treatments.

### Inclusion criteria

Patients of both sexes >18 year-old and with 12 months history of MA and with ≥2 attacks/month in the least 12 months, diagnosed according to ICHD-3 criteria, and upon treatment with NSAIDs (ibuprofen or diclofenac or nimesulide) were eligible for the study.

### Exclusion criteria

Hypersensitivity to study drugs, progressive serious clinical conditions (cancer, chronic hepatitis, human immunodeficiency virus), neuropsychiatric diseases (e.g., psychosis and depression, for the risk of low compliance), renal diseases (serum creatinine concentration more than 1.2 times the upper limit of the normal range according to the central laboratory reference values) and liver dysfunction (serum alanine or aspartate transaminase concentration more than 1.5 times the upper limit of normal range according to the central laboratory reference values). Patients with disorders capable of inducing the development of aura (i.e., patent foramen ovale, ischaemic stroke, restless legs syndrome, Parkinson's disease, and psychiatric disorders), patients with other diagnosis of headache (e.g., tension-type headache) and patients who did not sign the informed consent were also not considered eligible for the study.

### Sample

The study sample includes 20 patients with MA, 8 male (33–56-years, average 45.8 ± 7.8) and 12 female (19–61-years, average 38.5 ± 11.9). All enrolled patients received a daily treatment with umPEA (1,200 mg/day) for 90 days and used a NSAID (ibuprofen, 600 mg as requested and up to 1,200 mg; diclofenac sodium, 50 mg as requested and up to 100 mg/day, and nimesulide, dosage 100 mg as requested and up to 200 mg/day) in presence of acute headache pain.

Moreover, 20 patients with MA 10 male (35–59-years, average 42.4 ± 8.5) and 10 female (19–60-years, average 37.3 ± 10.6) were also enrolled in this study as positive control-group receiving a treatment with NSAIDs alone (ibuprofen, 600 mg as requested and up to 1,200 mg; diclofenac sodium, 50 mg as requested and up to 100 mg/day, and nimesulide, dosage 100 mg as requested and up to 200 mg/day) in presence of acute headache pain.

In both groups, the follow-ups were performed at 30 (T1), 60 (T2), and 90 (T3) days after the starting from the time of enrollment. Moreover, patients enrolled in these groups did not receive any prophylaxis treatment for MA.

### Assessment of efficacy

In agreement with our previous study (18), a visual analogical scale (VAS) was used to measure pain intensity before and after the pharmacological treatment. A total VAS summary score was calculated for each individual, adjusted, and reported on a 0–100 scale. Lower scores were associated with less pain and better function.

### Assessment of safety

Safety was assessed by monitoring drug-drug interactions and the incidence of adverse drug reactions (ADRs) which were assessed for both severity and causality, in agreement with our previous studies (19–22).

### Efficacy end-points

The primary efficacy end-point was defined as a statistically significant difference (*P* < 0.05) in the improvement of pain after um-PEA treatment measured during the three follow-up visits (T1–T3) compared to admission (T0). Another primary efficacy end-point was the improvement of disability (evaluated as the reduction of days with headache) after um-PEA treatment measured during the follow-up visits (T1–T3) compared to admission (T0). The secondary efficacy end-point was assessed measuring the reduction of NSAIDs consumption (ibuprofen, diclofenac sodium, or nimesuilde) in enrolled patients.

### Safety end-points

The primary safety end-point was defined as a statistically significant difference (*P* < 0.05) in the development of any adverse drug reaction. The secondary safety end-point was the development of drug-drug interactions during the study.

### Experimental protocol

For ruling out secondary headache, patients underwent a neurological examination, clinical biochemistry panel and radiological evaluation (X-ray, computed tomography and magnetic resonance imaging). Additionally a questionnaire was administered in order to confirm the clinical diagnosis of MA, according to ICHD-3 criteria (5), then a VAS was also administered.

All patients enrolled in umPEA-group received a daily treatment with umPEA (1,200 mg/day) for 90 days (end of the study); during the study, an add-on treatment with NSAIDs (ibuprofen, diclofenac sodium, or nimesuilde) was used for pain relief during acute migraine attack (about 2 days for each attack). Both umPEA and NSAIDs were bought by patients over the counter from the open market.

Patients enrolled in control-group did not received umPEA using ibuprofen or diclofenac sodium or nimesulide alone as symptomatic treatment.

The follow-up visits were performed, in all groups, at 1 (T1), 2 (T2), and 3 (T3, end of the study) months after the first administration of umPEA.

### Statistical analysis

A 17% difference in the VAS score was considered as minimal clinical improvement threshold. In order to assess the clinically relevant difference between each group, almost 20 subjects were enrolled in each group (power >80%, alpha 0.05, two-tailed). All data are expressed as mean ± standard deviation (SD). Data were checked for normality using the Kolmogorove–Smirnov test, while the Student's *t*-test was used as post-hoc test. The differences between multiple means was assessed using one-way ANOVA and the Kruskal–Wallis test. A multivariate analysis for age (continuous), sex (categorical), VAS score (continuous), and ADRs (continuous) was also performed. The threshold of statistical significance was set at *P* < 0.05. Statistical analysis was performed using SPSS software version 21 (SPSS Inc., Chicago, USA) was used.

## Results

### Patients

In all enrolled patients (umPEA-group and control-group), the laboratory parameters were in the normal range highlighting no systemic diseases. Neurological examinations and radiological findings confirmed the diagnosis of primary migraine excluding secondary causes, while the questionnaire confirmed the presence of aura.

All enrolled patients suffered from visual aura (with phosphenes and teicopsies) and severe pain (VAS 7–10; umPEA-group mean 8.0 ± 0.8: control-group mean: 8.05 ± 0.8, *P* = 0.288) (Tables 1, 2).

**Table 1:** Demographic characteristics of enrolled patients in umPEA-group at the time of admission.

**Sex**	**Age (years)**	**Attacks/month (*n*)**	**VAS (mm)**	**Days with pain during each attack (*n*)**	**NSAID**
F	19	2	8	4	Ibuprofen
F	24	2	9	2	Ibuprofen
F	48	4	9	3	Ibuprofen
F	61	4	7	4	Ibuprofen
F	38	3	7	3	Ibuprofen
F	48	4	8	3	Diclofenac
F	29	4	8	4	Ibuprofen
F	35	4	9	3	Nimesulide
F	49	3	8	3	Ibuprofen
F	30	3	7	4	Diclofenac
F	42	3	7	3	Ibuprofen
F	39	3	8	3	Ibuprofen
M	52	3	9	2	Diclofenac
M	38	4	9	4	Diclofenac
M	50	3	9	3	Nimesulide
M	56	4	7	4	Diclofenac
M	33	3	8	3	Ibuprofen
M	41	2	8	4	Ibuprofen
M	46	3	7	4	Nimesulide
M	50	3	8	3	Ibuprofen

*Data are expressed as mean ± standard deviation*.

**Table 2:** Demographic characteristics of enrolled patients in Control-group at the time of admission.

**Sex**	**Age (years)**	**Attacks/month (*n*)**	**VAS (mm)**	**Days with pain during each attack (*n*)**	**NSAID**
F	22	3	8	4	Ibuprofen
F	31	2	9	2	Ibuprofen
F	40	4	8	3	Ibuprofen
F	62	3	7	3	Ibuprofen
F	48	4	8	2	Diclofenac
F	32	4	8	3	Ibuprofen
F	31	4	9	3	Nimesulide
F	48	3	8	3	Ibuprofen
F	38	4	7	4	Ibuprofen
F	20	3	8	3	Nimesulide
M	55	3	9	3	Ibuprofen
M	35	3	9	4	Diclofenac
M	58	3	9	3	Nimesulide
M	43	3	8	4	Ibuprofen
M	56	3	7	3	Diclofenac
M	37	3	9	4	Ibuprofen
M	41	2	8	3	Ibuprofen
M	44	3	7	3	Nimesulide
M	28	3	7	4	Diclofenac
M	35	3	8	3	Ibuprofen

*Data are expressed as mean ± standard deviation*.

As shown in Tables 1, 2, at the time of enrollment (T0), in both groups, the patients showed about 3 attacks/month (umPEA-group mean: 3.2 ± 0.7; control-group male 3.15 ± 0.6, *P* = 0.333) and each pain attack lasted 2–4 days (umPEA-group mean 3.3 ± 0.7; control-group mean 3.2 ± 0.6, *P* = 0.27).

All enrolled patients (umPEA-group and control-group), did not receive any prophylactic treatment for MA, but during the acute headache attack used NSAIDs: ibuprofen (umPEA-group: 12 patients; control group: 12 patients), diclofenac sodium (umPEA-group: 5 patients; control group: 4 patients), and nimesulide (umPEA-group: 3 patients; control group: 4 patients) with no difference for sex or age (see Tables 1, 2).

As shown in Table 3, at T1 follow-up, umPEA treatment did not significantly affect pain headache (*P* = 0.0675), in both sexes, whereas a dramatic improvement in pain symptom was observed at T2 patients, and this effect was maintained at the last follow up (T3), irrespective to gender.

**Table 3:** Effect of um-PEA on VAS score expressed by gender.

**Sex**	**T1**	***P-*value**	**T2**	***P*-value**	**T3**	***P*-value**
Male	7.5 ± 1.1	0.06976	6.5 ± 1.6	0.01189	5.3 ± 2.5	0.0066
Female	7.9 ± 0.8	1	7.3 ± 1.1	0.006412	6.3 ± 2.5	0.01473

*Data are expressed as mean ± standard deviation. P-value for each follow-up (T1: 30 days; T2: 60 days; T3: 90 days) was calculated using the student t-test respect to T0 (admission) values*.

Moreover, at T3 both the days of pain and the number of attacks/months were significantly reduced (primary end-point) without difference for gender or age (T0: 3.1 ± 0.6; T3: 2.0 ± 1; *P* = 0.000, Table 3); a decrease in NSAIDs dosage was also observed (primary end-point; Table 4).

**Table 4:** Effect of 90 days um-PEA in enrolled patients.

	**Attacks/month Male**	**Attacks/month Female**	**Days of pain/attack male**	**Days of pain/attack female**	**Ibuprofen (mg/day)**	**Diclofenac (mg/day)**	**Nimesulide (mg/day)**
T0	2.9 ± 0.4	3.3 ± 0.8	2.85 ± 0.4	2.22 ± 0.6	1200	100	200
T3	1.5 ± 0.5	2.4 ± 1.1	1.5 ± 0.6	1.1 ± 0.3	600	50	100
*P*-value	0.000	0.005	0.000	0.000	0.000	0.000	0.000

*P-value was calculated using the student t-test respect to T0 (admission) values*.

In contrast, in control group, the treatment with NSAIDs alone, even if induced a significant decrease in the intensity of pain during each attack (*P* < 0.05), they failed to modify the pain intensity during the recurrence of attacks (T0: 3.15 ± 0.6; T3: T0: 3.1 ± 0.6, *P* = 0.164) or their number/month (T0: 2.85 ± 0.4; T3: 2.75 ± 0.4, *P* = 0.08; Table 5).

**Table 5:** ANOVA test analysis between case and control groups at admission (T0) and at the end of the study (T3).

	***F*-value**	***P***	**F statistic**
**ATTACKS/MONTH**			
T0	0.06	0.80	4.09
T3	16.93	0.000201	4.09
**VAS**			
T0	0.04	0.84	4.09
T3	12.91	0.000925	4.09
**DAYS OF PAIN**			
T0	0.25	0.62	4.09
T3	112.44	6.53E-13	4.09

*At T3 we documented a significant difference between control and umPEA- treated group in all variable evaluated (P < 0.01)*.

In both groups clinical examination and laboratory assays excluded the occurrence of major clinical event (see section Methods), therefore we excluded the incidence of side effects related to drug administration. All enrolled patients concluded the study and no patients were missing to the follow-up.

## Discussion

Migraine is recognized as a neurogenic disorder associated with secondary changes in brain perfusion (5). However, while the neuroinflammation affecting cranial blood vessels and dura sustains pain migraine in the early stages, the presence of allodynia, hyperalgesia, and expansion of nociceptive fields during migraine attacks is evocative of neuropathic pain, thus implying the involvement of further mechanisms, such as peripheral and central sensitizations (23, 24). As with other neuropathic conditions the therapeutic management of migraine is still a clinical challenge and several drugs have been proposed (7, 25), since their use may be related with the development of side effects (10, 25) or chronic migraine (26, 27).

In a previous case series, Hesselink (28) documented that the administration of PEA 1,200 mg/day in patients with neuropathic pain was able to induce a significant pain decrease. In agreement, we documented that the administration of topiramate and PEA was able to decrease pain symptoms in patients with nummular headache (15). Herein, we demonstrate for the first time that umPEA administration to patients with MA (1,200 mg/day for up 90 days) treated with common NSAIDs induced a significant pain relief (evaluated considering the VAS score and the number of attacks/month), irrespective to age or gender. These effects were evident at 60 days after the beginning of umPEA-treatment and lasted throughout the study. These results are in agreement with previous reports showing the anti-nociceptive action of umPEA in both preclinical models of neuropathic pain and with clinical trials performed in a variety of pain states (14, 23). The efficacy of PEA in reducing pain is related to its capability to interfere with the inflammatory mechanism within the nociceptive axis, allowing for a reduction of both peripheral and central sensitization. PEA activity encompasses both neuronal and non-neuronal cells (29, 30); the latter concerns the down-regulation of mast-cell hyperactivity mediated by this compound (31–33). Indeed, this cell population is often found in proximity to sensory nerve endings and through the release of inflammatory mediators and cytokines, stored in intracellular granules, they can enhance the nociceptive signal. Remarkably, mast-cells also colonize the spinal dura, the thalamus and the dura mater (31–33).

Moreover, in our study we also documented that patients treated with umPEA reduced the NSAIDs consumption, while this was not recorded in control-group.

In this frame, PEA might represent a useful therapeutic approach for migraine, as meningeal nociceptors can be activated locally through a neuro-immune interplay with resident mast cells populating the dura mater (34).

Previous studies reported that treatment with PEA does not cause adverse events or drug interactions and it doesn't induce pharmacological tolerance (16, 35–37). Our data demonstrate that um-PEA chronically administered for 90 days and occasionally added on to NSAIDs significantly reduces the score of pain intensity, the number of attacks/month, and the days of pain during each attack irrespective to age and gender, suggesting a synergic effect of these compounds.

This synergic effect is possibly related to the different mechanisms of action of the drugs used. In fact, PEA has dose-dependent anti-inflammatory and analgesic effects related to the modulation of mast-cell and microglia and is able to reduce pain, to preserve peripheral nerve morphology, to reduce endoneural edema, the recruitment and activation of mast cells, and the production of pro-inflammatory mediators (38–40). On the other hand NSAIDs have a dose-dependent anti-inflammatory and analgesic effect related to the inhibition of prostaglandins; however their use must be monitored, particularly in elderly patients, for potential gastrointestinal and hepatic risks, cardiovascular and renal side effects, and drug-drug interactions (41–43).

In our study, we did not record the occurrence of any major ADRs related to NSAIDs administration and this could be related to the short time of treatment (occasional use for 2 days) and also to the low dosage used. In particular, the treatment with um-PEA allowed a decrease in NSAIDs dosage in all patients suggesting that this combination may be useful to reduce the toxicity in patients underwent to polytherapy. The data also confirm the safety of treatment with um-PEA. Indeed, no adverse drug reactions or interactions were recorded during the study highlighting an optimal um-PEA pharmacological profile and the adherence with the umPEA regimen was good with a rate of 100%.

Finally, even without having performed a pharmacoeconomic analysis, these data suggest that this combination may help to reduce the cost of migraine, including drugs, hospitalization, and toxicity.

However, this study had some major limitations represented by the method used (single blind), the limited sample size and the brief duration of follow-up, so we defined this study as a pilot study and other clinical trials in a large population must be performed to confirm these data.

Although PEA is not reported in guidelines of migraine treatment, in our study the chronic administration of um-PEA to patients with MA in combination with NSAIDs, induced a significant pain relief allowing the reduction of the NSAID dosage. Remarkably, the compound was also able to reduce the number of migraine attacks. No major adverse drug reactions or interactions were recorded during the study. These data, compatibly with the design of the trial, are suggestive of an optimal pharmacological profile forum-PEA.

## Author contributions

DC, GD, and SS recorded clinical data. EC, MC, MW, TG, and NF analyzed clinical data. DC, LG, and VG conceived the study, had full access to all of the data, and wrote the manuscript. All authors have read and approved the final manuscript.

### Conflict of interest statement

The authors declare that the research was conducted in the absence of any commercial or financial relationships that could be construed as a potential conflict of interest. The reviewer RM and handling Editor declared their shared affiliation.
